# mHealth Tools for the Self-Management of Patients With Multimorbidity in Primary Care Settings: Pilot Study to Explore User Experience

**DOI:** 10.2196/mhealth.8593

**Published:** 2018-08-28

**Authors:** Anum Irfan Khan, Ashlinder Gill, Cheryl Cott, Parminder Kaur Hans, Carolyn Steele Gray

**Affiliations:** ^1^ Institute of Health Policy Management and Evaluation University of Toronto Toronto, ON Canada; ^2^ Department of Physical Therapy University of Toronto Toronto, ON Canada; ^3^ Bridgepoint Campus Lunenfeld-Tanenbaum Research Institute Sinai Health System Toronto, ON Canada

**Keywords:** primary care, mHealth, self-management, multimorbidity

## Abstract

**Background:**

Given the complex and evolving needs of individuals with multimorbidity, the adoption of mHealth tools to support self-management efforts is increasingly being explored, particularly in primary care settings. The electronic patient-reported outcomes (ePRO) tool was codeveloped with patients and providers in an interdisciplinary primary care team in Toronto, Canada, to help facilitate self-management in community-dwelling adults with multiple chronic conditions.

**Objective:**

The objective of study is to explore the experience and expectations of patients with multimorbidity and their providers around the use of the ePRO tool in supporting self-management efforts.

**Methods:**

We conducted a 4-week pilot study of the ePRO tool. Patients’ and providers’ experiences and expectations were explored through focus groups that were conducted at the end of the study. In addition, thematic analyses were used to assess the shared and contrasting perspectives of patients and providers on the role of the ePRO tool in facilitating self-management. Coded data were then mapped onto the Individual and Family Self-Management Theory using the framework method.

**Results:**

In this pilot study, 12 patients and 6 providers participated. Both patients and providers emphasized the need for a more explicit recognition of self-management context, including greater customizability of content to better adapt to the complexity and fluidity of self-management in this particular patient population. Patients and providers highlighted gaps in the extent to which the tool enables self-management processes, including how limited progress toward self-management goals and the absence of direct provider engagement through the ePRO tool inhibited patients from meeting their self-management goals. Providers highlighted proximal outcomes based on their experience of the tool and specifically, they indicated that the tool offered valuable insights into the broader patient context, which helps to inform the self-management approach and activities they recommend to patients, whereas patients recognized the tool’s potential in helping to improve access to different providers in a team-based primary care setting.

**Conclusions:**

This study identifies a more explicit recognition of the contextual factors that influence patients’ ability to self-manage and greater adaptability to accommodate patient complexity and provider workflow as next steps in refining the ePRO tool to better support self-management efforts in primary care ahead of its application in a full-scale randomized pragmatic trial.

## Introduction

Individuals with multimorbidity often require personalized, multifaceted self-management support to facilitate the acquisition of varying and evolving patient-centered goals [1-8]. Self-management is conceived as both an outcome, that is, positive health behaviors that individuals perform, such as exercise and medication adherence, as well as a process by way of the actions that individuals undertake to sustain health behaviors, such as goal setting, monitoring progress toward goals, and identifying barriers to self-management [1]*.*

Primary care providers play a central role in collaborating with patients to help them successfully manage their multimorbidity [9,10]. Supporting self-management efforts involves collaboratively helping patients and their caregivers acquire the skills and confidence to manage their chronic conditions and regularly assessing progress toward patient goals and addressing any challenges faced [11]. Providers in primary care settings have indicated that conflicting symptom profiles, uncertainty around treatment regimens, and the absence of guidelines around managing patients with multiple chronic conditions affects their ability to effectively support self-management in adults with multimorbidity [12]. This is further complicated by a number of challenges, including the compound effects of different conditions and medications, emotional strain, and diminished motivation that patients’ may experience as they try to manage competing health conditions and issues of social complexity such as low-income status and limited health literacy [13,14].

The use of mHealth is demonstrated to be an effective means to support self-management efforts to help mitigate the challenges that patients and primary care providers experience [10,15-19]. mHealth technology can enable the integration of self-management support into an individual's daily routine by allowing them to access educational materials, record health behaviors, track health data (blood glucose or blood pressure readings) on an ongoing basis and share that information with their primary care providers [20] to allow for collaborative management of their chronic conditions [21]. The adoption of mHealth tools in primary care settings has received considerable attention in the literature, given the central role that the primary care sector plays in supporting community-dwelling adults with chronic conditions [10,22,23]. To date, however, much of the existing exploration around the influence of mHealth apps in facilitating self-management has focused on patient experience involving a single chronic condition, such as diabetes [24,25], hypertension [26], asthma [27,28], and chronic obstructive pulmonary disease [29], with provider perspectives receiving considerably less attention [30,31].

As such, our understanding of the effectiveness of mHealth apps in supporting self-management and their impact on outcomes in adults with multiple chronic conditions remains in its early stages [3,32]. In light of the growing prevalence of multimorbidity among patients being managed in primary care settings [33-35] and the unique challenges in self-management faced by individuals with multimorbidity [8], a more targeted effort toward exploring the development, uptake, and outcomes associated with using mHealth apps to support self-management in this particular patient group is increasingly needed.

The electronic patient-reported outcomes (ePRO) tool is among a small number of mHealth tools developed using a user-centered design approach [36] with an explicit focus on facilitating self-management in patients with multiple chronic conditions. An initial usability assessment of the ePRO tool was conducted, and findings suggested relatively low usability and feasibility of the ePRO tool [37]. To build on the results obtained from the usability pilot, this paper aims to examine patient and provider views and experiences pertaining to the use of the ePRO tool to better understand how the tool supports self-management among patients with multimorbidity. Our analysis was informed by the Individual and Family Self-Management Theory (IFSM), a well-recognized theory that highlights key aspects of successful self-management, which has previously been used to assess self-management efforts aided by technology [38,39]. IFSM posits 3 constructs related to self-management including contextual aspects, that is, individual and external factors that might challenge or enhance the patient’s ability to successfully self-manage, and the provider’s capacity to support self-management efforts; procedural aspects of self-management, including self-regulation (goal setting, self-evaluation, and self-monitoring), self-efficacy, and social facilitation (social support for self-management); and proximal or short-term and distal outcomes such as changes in health behaviors, reduced health system utilization, and costs [40]. Multimedia Appendix 1 presents a visual depiction of the IFSM theory.

## Methods

### Design

This study was based on a secondary analysis of data from a usability pilot of the ePRO tool and sought to assess the user (patient and provider) experience of the tool in supporting self-management for patients with multimorbidity in an interdisciplinary primary care team.

### Setting and Participant Recruitment

At the time of the study, the primary care team was composed 5 family physicians, 1 nurse practitioner, 1 social worker, 2 registered nurses, 2 medical assistants, 2 diabetes educators, and 6 administrative staff members. Patients had a lead provider (physician or nurse practitioner) that managed care, although patients could also receive care from other providers (social worker and dieticians) on the primary care team [41]. The focus of this study was patients with multiple chronic conditions and social complexity.

Provider recruitment was initiated through the managing director of the primary care team who was asked to identify providers that would be interested in participating, given the tool’s intended functionality and focus on patients with multimorbidity. A summary of the research study and consent form was sent to those providers identified by the managing director. Following the completion of consent forms, providers from the primary care team shared the names and contact information of potential participants with the research team.

Patient participants had to meet the following eligibility criteria [37]: 1) patient was considered to be multimorbid, which could include physical chronic illness as well as social complexity and mental health issues, and 2) they had the physical capability to use a tablet or have a caregiver with the physical capability to use a tablet to input data on behalf of the patient. Eligible patient participants were contacted directly by the primary care team’s administrative staff over the phone or when they checked in for appointments. Patients were informed of the study and provided consent to be contacted by the research team if they were interested in participating [37]. Patients provided informed consent to participate in the study by signing consent forms when they received training on how to use the ePRO tool before starting the study. Patient information, including age, comorbidities, birthplace, and comfort with technology, was collected at the outset of the study to provide contextual information for later analyses. Furthermore, full ethics approval for recruitment was obtained and data were collected from the Joint Bridgepoint Hospital-West Park Healthcare Centre-Toronto Central Community Care Access Centre-Toronto Grace Health Centre Research Ethics Board.

### Electronic Patient-Reported Outcomes Tool Features

The ePRO tool was developed by a group of primary care providers and patients in an interprofessional primary care team based in Toronto, Canada; a detailed description of the development process has been published elsewhere [37,42]. The tool includes a mobile platform that is linked to a Web-based portal system and has 2 key components (1) goal setting to develop and track self-management goals, and (2) a hospital checkout function to notify providers of hospital visits. Goals were focused on 5 key themes: physical and social well-being, mental well-being (specifically mood and memory), mobility, pain management, and weight/diet management. Goal themes were linked to a designated monitoring protocol based on indicators from a variety of validated scales, including the Patient-Reported Outcomes Measurement Information System (PROMIS) Global Health Scale, PROMIS Pain Interference Scale, PROMIS Health Assessment Questionnaire, Generalized Anxiety Disorder Scale, and the Patient Health Questionnaire, which are considered to be valid and reliable in the context of patients with chronic conditions [43-45]. The weight and diet theme allowed patients to take photos of their food and track their weight on an ongoing basis. In addition, the tool had a hospital checkout feature that allowed patients to notify their provider when they had visited and been discharged from a hospital. Of note, the ePRO system is compliant with the relevant Canadian and American health information and security laws. All system use data from the mobile phone app and web portal was linked to a secure server, which was managed by the technology partner QoC Health Inc (Toronto, Canada), and a confidentially agreement was also completed by QoC Health Inc.

### Training

The technology partner (QoC Health) offered providers two 1-hour hands-on training sessions (facilitated by the research team) on the mobile phone app and portal to provide a walk-through of the ePRO tool before starting the study, whereas, patients received one-on-one training through a 30-minute hands-on session with a member of the research team at the time when patients gave consent to participate in this study.

### On-Boarding and Electronic Patient-Reported Outcomes Tool Use

Each participating patient received a Samsung Core mobile phone (including 3G data coverage) that had the ePRO mobile app preloaded. In addition, patients had the option to enter monitoring data through the portal instead of the mobile device. Providers could access the desktop version of the app through the portal system, which allowed them to collaboratively establish goals with patients, track patients’ progress over time, and access the hospital checkout alerts. The portal system was generally expected to be used primarily by providers when establishing goals and to review patient progress.

At the outset of the study, patients and providers met to collaboratively determine what goals (and associated monitoring protocols) they wanted to establish and track over the course of the study. During the initial 30-minute consult, patients and providers discussed their existing and evolving needs as they relate to managing their multimorbidity, collaboratively established goals, and identified relevant monitoring protocols pertaining to their self-management goals. After this initial meeting, patients tracked their progress toward goals for 4 weeks using the mobile phone app or the portal, following which they met with their provider to view their results and discuss progress to date. Furthermore, patients could see their providers on a more frequent basis if needed (and discuss results or adjust goals, etc), but the initial set-up meeting and final results debriefing were mandatory for all participants.

### Data Collection and Analysis

Upon completion of the 4-week study period, patients and providers were invited to participate in separate focus groups involving semistructured questions to offer feedback on their experience. Patients were asked whether the tool addressed pertinent issues, what improvements could be made, and whether they felt the tool was easy to use. The patient focus group (PTFG) was conducted in December 2014 and moderated by a member of the research team (CSG). A total of 5 patients participated in the PTFG, which lasted 75 minutes. In addition, 3 patients were unable to attend the PTFG and were interviewed separately (interviews lasted 45-60 minutes). Conversely, the provider focus group (PRFG) was conducted by CSG in January 2015 (lasted 60 minutes) and included all 6 providers who participated in this study. Providers were asked about the ease of use, whether they could incorporate the tool into the workflow, and what improvements could be made.

Initially, members of the research team (AIK, AG, CSG, and PH) independently reviewed transcripts and then met iteratively to achieve consensus around a common coding scheme [46]. Following the completion of the qualitative descriptive stage where common themes were identified using a thematic content analysis approach [46], 2 members of the research team (AIK and AG) individually mapped emerging themes pertaining to patient and provider experiences of using the ePRO tool onto the context, process, and outcomes domains of the IFSM theory [40] via the framework method for qualitative data analysis [47] to better understand how the ePRO tool supports each of these components in facilitating self-management for adults with multimorbidity. Following this initial conceptualization, the authors held a consensus meeting to address any gaps or discrepancies in the mapping procure. A description of the IFSM’s core domains is available below:

1. *The context* domain encompasses individual, family, and environmental factors that may challenge or enhance a patient’s ability to successfully self-manage and provider capacity to support self-management efforts [40]. Contextual factors pertaining to both patients and providers who participated in this study were identified through interview and focus group transcripts.
2. *The process* domain refers to procedural aspects of self-management, including self-regulation (goal setting, self-evaluation, and self-monitoring), self-efficacy (which refers to the degree of confidence an individual has in their ability to adjust or alter behavior successfully), and social facilitation (ie, social support for self-management and the interplay between the perspectives of patients, families, and providers on goals and techniques for self-management) [40]. Patient and provider feedback was assessed using this process lens to explore the tool’s role in supporting or inhibiting key self-management processes.
3. The *outcome* domain includes short-term outcomes, such as successful symptom management and changes in health behaviors, which over time might result in distal outcomes, including reduced health system utilization and costs [40]. Interview and focus group transcripts were reviewed to explore patient and provider perceptions of outcomes pertaining to their experience of using the ePRO tool over the duration of the study.

## Results

### Sample Characteristics

A total of 12 patient participants were identified and agreed to participate in this study. Over the course of the study, 3 patients dropped out owing to health concerns. Patient participants were on average aged 58 years (range 35-72 years) with an equal ratio of males to females. Patients presented with both medical and social complexity—a typical patient in the sample could be described as a female aged 58 years with multiple chronic conditions, including hypertension, chronic obstructive pulmonary disease, arthritis, and depression. Half of the patient group (n=6) reported feeling comfortable with hand-held devices and technology. Most patients tracked one or two goals, and physical health was the most commonly tracked goal. In addition, 6 providers participated in this study, including family physicians, a registered nurse, dietician, social worker, and diabetes educator. Table 1 presents a summary of patient characteristics and system use. The key themes that were drawn from the PRFG and PTFG as well as interviews were mapped onto the core domains of the IFSM framework and are summarized in Table 2.

### Self-Management Context

Contextual factors that both user groups identified as important considerations in developing mHealth tools for self-management included the expansion of the tool’s scope to allow for comprehensive self-management and linkages with programs and services offered at the practice. In addition, providers described a gap between the tool’s design and provider workflow and an underlying disconnect between the goals established at the outset and the monitoring protocols available in the tool, indicating that many questions lacked specificity and were not adequately personalized to align with individual patient context.

#### Patient Views

Patient interviews indicated that they perceived the ePRO tool as a means to manage their conditions and health behaviors more holistically and as such, they felt the tool had limitations in terms of the comprehensiveness of its scope. Given the wide range of conditions and symptoms patients are attempting to monitor and regulate as part of their self-management efforts, features such as appointment and medication reminders, blood sugar reporting, pedometer functionality, and links to other mHealth apps (such as Sugars and FitBit) were identified as necessary in expanding the tool’s scope to reflect the broader individual context within which patients were hoping to self-manage their conditions and symptoms. In addition, patients stressed the need for greater personalization and customizability of goals and monitoring protocols. The questions in the ePRO tool appeared to lack the depth that was considered vital to incorporating patient context into self-management activities:

For the mood one, the questions I got asked...would have been okay for somebody that didn’t have much of a mood problem.

Patient 05, PTFG

Not everybody needs the same questions. Everybody's case is different...but we got the exact same questions.

Patient 11, PTFG

I think the bottom line that comes out of my disconcertedness about it was that the questions were too broad. They weren't specific enough.

Patient 09, Interview

**Table 1:** Participant characteristics.

Characteristics		Value
**Age (years), n (%)**		
	35-54	5 (46)
	55-65	4 (36)
	>65	3 (27)
**Sex, n (%)**		
	Female	6 (50)
	Male	6 (50)
**Reported ease with technology, n (%)**		
	Previous experience	6 (50)
	Little experience	2 (17)
	Not reported	4 (33)
**Chronic conditions, n (%)**		
	Arthritis	2 (17)
	Cardiovascular	3 (25)
	Chronic pain	5 (42)
	Diabetes	5 (42)
	Mental health	10 (83)
	Obesity	2 (17)
	Renal failure	2 (17)
	Chronic obstructive pulmonary disease	1 (8)
	Other	2 (17)
**Goals tracked, n (%)**		
	Physical health	6 (50)
	Mood and memory	3 (25)
	Pain	2 (17)
	Diet	2 (17)
	Mobility	1 (8)
**Questions per module and number of unique completions^a^**		
	Physical health	9-76 completions
	Mood and memory	8-70 completions
	Pain	8-53 completions
	Diet	3-9 completions
	Mobility	25-3 completions
	Hospital alert	2-1 completions

^a^Unique completions refers to the frequency of times patients completed a full report of protocol questions.

**Table 2:** Summary of themes observed across user groups.

IFSM^a^ domains		Thematic mapping	Key themes	
			Patient views	Provider views
1.	Context: Individual, family, and environmental factors that might challenge or enhance patient ability to successfully self-manage and provider capacity to support self-management efforts	Contextual aspects pertaining to users (patients and providers) that inhibit (or facilitate) self-management efforts	Expansion of tool scope to promote comprehensive self-management Need for improved alignment between the tool’s design and patient complexity	Linkage with existing programs and practice approaches Tool responsiveness to challenges presented by self-management in patients with multimorbidity
2.	Process: Procedural aspects of self-management, including self-regulation (goal setting, self-evaluation, and self-monitoring), self-efficacy (ie, the degree of confidence an individual has in their ability to adjust or alter behavior successfully), and social facilitation (eg, social support for self-management and the interplay between the perspectives of patients, families and providers on goals and techniques for self-management)	ePRO^b^ tool’s role in supporting or inhibiting key self-management processes	Limited progress toward self-management goals further inhibits a patient’s sense of self-efficacy Need for more active feedback and support from providers for self-regulation activities	Challenges in promoting self-efficacy for patients with multimorbidity Concerns with offering active social support through the tool
3.	Outcomes: Proximal or short-term outcomes, such as changes in health behaviors, as well as distal outcomes, such as reduced health system utilization and costs	The impact of the ePRO tool on self-management efforts—patient and provider perspectives on the tool’s effectiveness	Capacity building for expanded self-management support across providers in the primary care team	Key insights into broader patient context and its underlying impact on patient ability to self-manage

^a^IFSM: Individual and Family Self-Management Theory.

^b^ePRO: electronic patient-reported outcomes.

#### Provider Views

Providers acknowledged the challenges that multimorbidity presents for patients engaged in self-management and suggested expanding the tool’s capabilities to account for how complexity attributed to multimorbidity dominates decisions around which self-management strategies providers adopt in caring for these patients. Providers emphasized the need for greater connectivity with existing mHealth apps and services offered by the primary care practice:

Align it with...programs that we're doing already. Like Her Story or She Rose, Craving Change...the Stop Study...Every single one of the patients that [are] coming... to see me is very complicated–medically, socially and psychologically...Smoking cessation, changing your behavior around eating, tracking food and how you're feeling...would align [with patient needs]...and those are already people, that are engaged in the sense that they are coming to those groups.

Provider 04, PRFG

Providers noted that individual complexity profiles along with variability in patient readiness for behavioral change requires a flexible approach toward self-management. As such, the adoption of the ePRO tool might not be appropriate for all patients. In fact, one provider offered the example of a patient who they felt was not ready for an active self-management intervention.

If I had been able to think about it more in advance or understood this better in the beginning, I would not have chosen that participant because he needed a lot more motivational interviewing to get to the point where we could do that kind of goal setting. He wasn't an ideal candidate for this kind of test. But it was very difficult for him...he found having to look at that information depressing...all of which points to the fact that he was not ready to set those goals.

Provider 05, PRFG

Several providers revealed that the ePRO tool did not match existing approaches to goal setting used in the primary care practice. Providers were accustomed to using the “specific, measurable, attainable, realistic, and timely” (SMART) goals approach [48] to guide goal setting efforts and expressed that the format and process for goal setting built into the tool was not aligned with the SMART framework. Multiple providers expressed that this disconnect along with limited connectivity with electronic medical records restricted the extent to which they could adopt the tool as a standard part of care.

[The tool] has to be tailored to work flow, I would like that the visit could be somehow linked into our electronic medical record. Because I was documenting...I was like dealing with the [ePRO] template and setting the goal. But then that doesn't document it in the patient's chart.

Provider 04, PRFG

Although providers indicated that the tool could be a useful component in supporting self-management in this patient population, self-management for patients with multimorbidity is an inherently long-term and multilayered process due to the compounding of medical and social complexity that commonly occurs in this patient group. As one provider stated:

...What I usually do with people is just layers and layers and layers and layers of therapy...And you can't even get to the therapy because... they're at risk for homelessness. Then they're not getting their Ontario Works cheque. And so you're just kind of going along and along. It takes me a long time to meet with somebody once you get through all of that stuff and then go, “Let's set some goals.”

Provider 06, PRFG

### Self-Management Processes

Patient views pertaining to how the ePRO tool supports self-management processes revolved around the patient’s desire for more active engagement and support from providers directly through the tool and how limited progress toward self-management goals detracted from the patient’s sense of self-efficacy and self-regulation activities. However, providers indicated concerns with offering active social support directly through the tool and discussed the challenges that multimorbidity presents in promoting self-efficacy.

#### Patient Views

Several patients described how monitoring their limited progress toward goals through the tool actually led them to feel discouraged and demotivated, thereby hindering confidence in their ability to self-regulate and meet intended self-management goals. As several participants noted:

...It's going to take weeks before I might move up to the next level. All you're doing in that case for me is reminding me that my fitness...is crappy... If I had to answer that question every day that my fitness was bad, it's not motivating. It's sort of sinking into my head that my fitness is crappy...It's continuing to be crappy. So depending on the person that might get you depressed.

Patient 012, PTFG

You know, you go up and down. I mean even in a day, I could go down, you know. And I was told, you know, just put it in at nighttime...But you know, if I put it in at nighttime, probably from night to night, it would change if I just rate it at that minute. But throughout the whole day...there was nothing to say, you know, I'm on a total rollercoaster ride.

Participant 05, PTFG

Patients identified gaps in the tool’s ability to promote self-efficacy in terms of the adherence to self-regulation activities because of limited feedback on their progress from providers. Patient-reported data were not reviewed by providers in real time nor were providers able to comment on patient progress directly through the tool; this gap in functionality led to patients feeling isolated and discouraged.

The problem is I don't know what the picture [refers to uploading photos of meals consumed to better track their diet to manage their diabetes] is doing. Like there's no one analyzing the picture—is there?

Patient 012, PTFG

Patients suggested the use of alerts in the form of reminders (that could be sent by the provider or programmed into the ePRO tool) for logging data to support self-regulation. Patients also suggested that providers use the tool to offer direct encouragement and acknowledgment of patients’ progress and monitor patient-reported data in real time (and intervene in a timely manner if needed). As one patient expressed:

Your provider should be able to trigger an event because you weren't answering a question...But there's no ability to...I got no feedback from my provider until today. And it was useful feedback...So how do you keep up on your goal? Because the onus was on you

Patient 012, PTFG

Patient expectations around social facilitation and social support indicated that they were expecting a more active role in self-management efforts from providers. Patients felt the tool should supplement patient-provider interaction through regular feedback and encouragement as an “add-on” to existing in-person appointments rather than a replacement for in-person interaction and consults with their providers.

#### Provider Views

Provider interviews indicated that careful attention and adjustment is vital in ensuring that self-regulation activities and subsequent monitoring protocols are appropriately selected. Limited progress toward self-management goals inhibits self-efficacy and leaves patients feeling unmotivated and unlikely to continue with self-regulation and monitoring activities. As one provider explained:

A lot of times when people set goals, it doesn't work, especially the first time or couple of times...A lot of people set goals that are way too big, way too out there, or not realistic until they actually apply it...So then that sets them up for dropping out maybe. Because they're like, oh, I can't do this goal, I failed, I suck.

Provider 04, PRFG

Providers also indicated that self-management is a fluid process and as such, the tool must be more responsive to the changing self-management needs of this patient subgroup. As one provider said:

For concrete goals, this can be a useful tool. Patients who can set those kind of concrete goals can implement them. They probably don't need the tool...Most of the goals I set are not like that...they'refluid and they change. And I didn't find this tool to be responsive enough to offer that.

Provider 05, PRFG

In contrast to patient views, provider expectations pertaining to how the tool would be operationalized in the daily practice were centered on the tool’s potential in remotely monitoring patient progress. Providers envisioned the tool being used to flag problems or lapses in patient adherence to self-regulation activities (rather than a mechanism for active social facilitation of self-management efforts), such that providers only intervene as needed, thereby reducing the need for in-person appointments. As one provider noted:

One of the things that I do is tell people who are initiating insulin to increase their dose. And I have to be in regular contact with them a lot. Like way too much. If this tool could tell me the information, they could just punch it in—what their blood sugar was that day, how much insulin they took. And the only time we would have to be in touch would be if there was a problem until there was regular follow-up scheduled. That would be perfect.

Provider 05, PRFG

Providers had concerns about active engagement and communication with patients through the tool; in particular, they highlighted challenges with information overload related to reviewing patient-reported data in real time and associated liabilities from a medical and legal perspective. Overall, they envisioned the tool as more of a means to allow for remote monitoring (when patients readiness to self-manage was evident).

### Proximal or Short-Term Outcomes

Patient and provider views on the ePRO tool’s impact on enabling self-management efforts indicated that both user groups felt that the tool had made important contributions in terms of proximal or short-term outcomes pertaining to successful self-management. Patients described the tool’s potential in enabling wider support for self-management from multiple providers in the primary care team. Conversely, providers stated that the tool offered important insights into the contextual aspects that inhibit a patient’s ability to engage in self-management activities. Specifically, access to patient-reported data, that is, mood, health behaviors, etc, had helped them to explore trends in self-regulation and goal attainment over time and more easily identify the underlying motivations and barriers that patients may face in self-management.

#### Patient Views

Patients acknowledged the potential of the ePRO tool in building capacity to support self-management in a team-based care environment by helping to better distribute the workload across providers to meet the evolving needs of patients. As one patient expressed:

It could be really valuable in terms of...balancing the time of those professionals in a way that could help the patient...with that goal but also manage the time of the professionals on the team in the most appropriate way...Instead of someone saying I need to see my doctor every week...as you're seeing those results, you could see, well, basically the issue here is with what I'm eating. I need to talk to the dietician. So they wouldn't be going back to the physician every week. You would have other people stepping in when they would see from the tracking that you needed support.

Patient 02, Interview

#### Provider Views

Providers emphasized the value of the ePRO tool in helping to generate insights into underlying patient context (ie, patient preferences and readiness) to offer a fulsome sense of how patients are coping, and thereby adjust goals and self-management activities as needed. One provider describes how data obtained through the tool offered a sense of the challenges that her clients face, and this helps to contextualize overall progress toward goals and allows for the timely identification of barriers to self-management:

When [the patient] was experiencing a lot of pain...the other goals drop off because she's not going to be attempting those. And even if [the patient] hadn't linked those then we could have linked them at the visit. But [the patient] had already obviously linked those two things together. So it was kind of nice to have that pain data, to see that...

Provider 02, PRFG

## Discussion

### Principal Findings

In light of the growing prevalence of individuals with multimorbidity being managed in primary care settings [1], the development, refinement, and evaluation of mHealth tools to better assist patients and primary care providers in effectively self-managing chronic conditions is an important research priority [17]. Findings indicated that both patients and providers recognized that the nature of self-management support a patient requires varies based on their medical and social complexity as well as readiness for change. As such, both user groups expressed the importance of greater alignment between tool capabilities and underlying self-management context in light of the complex and evolving nature of self-management needs for patients with multimorbidity. In addition, providers emphasized the need to recognize practice context and better integrate the tool into workflow (ie, linkage with electronic medical records and consistency with goal-setting procedures used in practice such as the SMART goals framework). Some dissonance emerged between the expectations of patients and providers around the role of the tool in providing social support for self-management processes, wherein providers see the tool as a possible mechanism to reduce in-person appointments and patients expect more active feedback, social support and facilitation from providers through the tool. Both patients and providers acknowledged the tool’s value in supporting self-management efforts; patients felt that the tool could help to better distribute the workload across providers in an interdisciplinary team setting, and providers acknowledged that the tool offers critical insights into how patient context influences progress toward goal attainment and helps inform provider approaches toward self-management.

Connectivity with existing mHealth apps (ie, fitness tracking and blood sugar monitoring) was strongly emphasized by both patients and providers, which is an important finding, given that among patients managing multiple conditions [8], the disproportionate effect of a single condition can adversely impact their physical or psychosocial functioning and capacity to self-manage [3,6,14]. As such, both user groups highlighted the potential of the ePRO tool in serving as a comprehensive hub for self-management support where they could conceivably input data from other sources (ie, other mobile phone apps, etc) to manage multiple goals and track symptoms and progress over time.

Patients and providers both highlighted the need to adopt a more patient-centered approach for self-management activities (ie, monitoring protocols offered in the tool) for greater alignment between the tool’s functionality and individual patient context, citing a disconnect between patient goals and the monitoring protocols available in the tool and emphasizing the need for more personalized content [49,50] and realistic goal setting [51]. Moreover, these findings reflect existing research, which has found that mHealth solutions are likely to have greater uptake when they allow for personalization [52] and can be integrated into workflow [30,53]. In addition, proactively identifying and adjusting patients’ expectations around the pace of self-management progress and challenges must be considered when determining whether the tool is an appropriate fit [16]. Fit is particularly important for individuals with multimorbidity, who often experience symptoms that may be interdependent and impart a compounded effect on their ability to self-manage [3,8,54-56]. As such, the use of mHealth tools to support self-management in patients with multimorbidity must reflect the complexity and inherent variability in each patient’s experience of multimorbidity [55].

From the perspective of providers, improving the tool’s alignment with current workflow (such as the incorporation of the SMART goals approach) and integration with electronic health records were key aspects of suggested improvements, indicating the need for proactive consideration of organizational context and provider workflow when developing and implementing mHealth tools [16,53,57,58]. In addition, ensuring that providers have adequate time to acquire training on the tool’s functions [59,60], minimizing clinical and workflow redesign [30,60], and financial compensation for the extra time and effort required of providers to incorporate mHealth tools into standard practice are key considerations in improving uptake and acceptance among providers [60,61]. Furthermore, although providers suggested linkages with electronic medical records to improve the tool’s functionality, issues pertaining to breaches of privacy, secure transmission of data, devices getting lost or stolen, and user data being accessed in an unauthorized or unsecure manner must also be considered [62,63].

In this study, both user groups recognized that goal setting and self-management is a fluid process that varies according to a patient’s social and medical complexity and readiness [3,8,64]; thus, self-management processes, including decisions around goal setting and the selection of monitoring protocols, must be adaptive to that complexity [6,65]. Providers acknowledged the ePRO tool’s potential in identifying important contextual information, including patient readiness, preferences, and barriers to self-care, and also emphasized the importance of understanding the impact of these contextual factors on influencing the patient’s ability to self-manage [57,66,67]. Compared with previous research, which found that providers tend to view self-management from a biomedical lens with a strong emphasis on individual responsibility [68], in this study, both user groups recognized how medical, psychological, and social aspects collectively inform patient ability to self-manage.

Patient feedback indicated they wanted providers to have a more “active” presence through the ePRO tool; the suggestions included push factors, such as regular feedback and encouragement from providers, and alerts if providers observe deviations in results, which is consistent with other studies of self-management using mHealth tools [17,69-73]. Patients viewed the tool as a supplement rather than a replacement to existing care, which resonates broadly with patient perspectives on the role of eHealth tools in influencing care [65,73,74]. This particular finding also reflects previous research on patients with multimorbidity and their preferences around collaborative care management involving primary care providers, which found that participants indicated a willingness to use technology for monitoring or educational purposes if it did not eliminate or inhibit human contact with their providers [54]. Findings suggested that patients expressed fears of feeling isolated or being abandoned (by their providers) as a function of adopting a mHealth tool for self-management, which is also consistent with previous research in this topic area [73].

In contrast, providers perceived the ePRO tool as a way to enable remote monitoring of patients and intervene as needed, highlighting an important disconnect in expectations between the 2 user groups. This observation differs somewhat from previous research, which found that providers are often concerned about placing too much responsibility around self-management on patients without adequate guidance, emphasizing the need to strike a balance between patient autonomy and provider support [51,58]. Furthermore, this finding warrants the importance of examining how technologies to support self-management are developed in terms of whether they promote a patient-driven or provider-centered view of self-management and being aware of possible dissonance between user groups to adjust the tool accordingly [66,70].

### Limitations

The challenge of small sample sizes is a common aspect of many studies focused on pilot-testing of mHealth interventions [75]; thus, we cannot make generalizations about observed findings. A small number of dropouts occurred during the study, highlighting the challenges of working with patients with multimorbidity and issues of attrition that are commonly observed in mHealth and Web-based interventions [76-78], including interventions involving self-management [78,79]. However, in alignment with past formative work around the development of mHealth tools, this study offers a detailed comparison of patient and provider perspectives around the use of a mHealth tool to support self-management with respect to a patient group with a unique set of needs. Owing to time and resource constraints, the provider on-boarding meeting was not observed by a member of the research team, which is an important limitation of our design. In addition, although proximal outcomes were identified based on the user feedback, clinical or long-term outcomes were not assessed. A randomized pragmatic trial is underway to examine the impact of the ePRO tool on clinical and system-level outcomes. Another limitation involves the challenges of working with a complex patient subgroup, which often involved dealing with issues regarding recruitment, including attrition and difficulty with follow-up, owing to challenges stemming from their medical and social complexity [80]. It can also be difficult to engage providers because of their limited availability, which can affect their ability to actively participate in development and testing phases despite their enthusiasm and interest.

### Conclusions

Past research has mostly focused on the use of mHealth tools for patients in a single disease group [29,53,81-84]. Multimorbidity can complicate approaches to self-management because of complex (and often conflicting) clinical practice guidelines and competing priorities that providers and patients often face [6]. Incorporating a user-centered design approach in developing and refining mHealth tools is critical to ensure alignment with underlying user context and processes pertaining to self-management support [25,85]. Broadening the tool’s scope to allow for greater customizability of content to enable personalized goal-oriented care [86], and the addition of features, such as secure communication, between patients and providers through the tool, and connectivity with other mHealth apps are some aspects that are under consideration in terms of refinement of the tool prior to a full-scale application in the upcoming randomized pragmatic trial.

## Appendix

Multimedia Appendix 1 Model of the Individual and Family Self-management Theory.

## Abbreviations

ePRO: electronic patient-reported outcomes
IFSM: Individual and Family Self-Management
PRFG: provider focus group
PROMIS: Patient-Reported Outcomes Measurement Information System
PTFG: patient focus group
SMART: Specific, measurable, attainable, realistic, and timely
