# Supplementary material for: mHealth Tools for the Self-Management of Patients With Multimorbidity in Primary Care Settings: Pilot Study to Explore User Experience
Source: JMIR Mhealth Uhealth. 2018 Aug 28;6(8):e171. doi: 10.2196/mhealth.8593 (PMC6134226; doi:10.2196/mhealth.8593)
Supplement: Multimedia Appendix 1 [file mhealth_v6i8e171_app1.pdf]

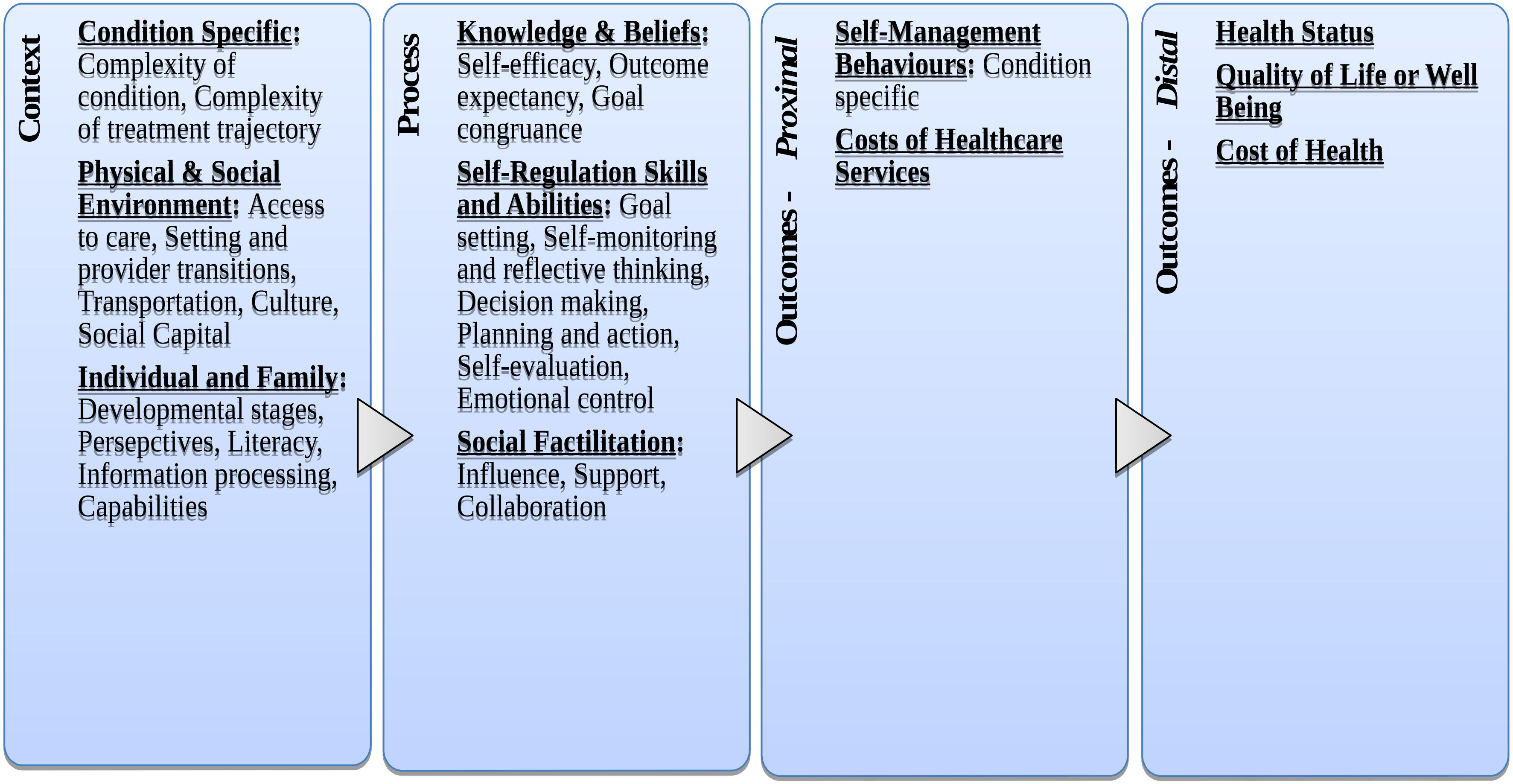

Appendix 1: Model of the Individual and Family Self-management Theory (Source: Ryan P, Sawin KJ. The Individual and Family Self-management Theory: Background and Perspectives on Context, Process, and Outcomes. Nurs Outlook. 2009;57(4):217-225.e6
